# Solitary median maxillary central incisor (SMMCI) syndrome

**DOI:** 10.1186/1750-1172-1-12

**Published:** 2006-04-09

**Authors:** Roger K Hall

**Affiliations:** 1 Department of Dentistry, Royal Children's Hospital, Flemington Rd Parkville, 3052 Victoria, Australia

## Abstract

Solitary median maxillary central incisor syndrome (SMMCI) is a complex disorder consisting of multiple, mainly midline defects of development resulting from unknown factor(s) operating *in utero *about the 35th–38th day(s) from conception. It is estimated to occur in 1:50,000 live births. Aetiology is uncertain. Missense mutation in the *SHH *gene (I111F) at 7q36 may be associated with SMMCI. The SMMCI tooth differs from the normal central incisor, in that the crown form is symmetric; it develops and erupts precisely in the midline of the maxillary dental arch in both primary and permanent dentitions. Congenital nasal malformation (choanal atresia, midnasal stenosis or congenital pyriform aperture stenosis) is positively associated with SMMCI. The presence of an SMMCI tooth can predict associated anomalies and in particular the serious anomaly holoprosencephaly. Common congenital anomalies associated with SMMCI are: severe to mild intellectual disability, congenital heart disease, cleft lip and/or palate and less frequently, microcephaly, hypopituitarism, hypotelorism, convergent strabismus, oesophageal and duodenal atresia, cervical hemivertebrae, cervical dermoid, hypothyroidism, scoliosis, absent kidney, micropenis and ambiguous genitalia. Short stature is present in half the children. Diagnosis should be made by eight months of age, but can be made at birth and even prenatally at 18–22 weeks from the routine mid-trimester ultrasound scan. Management depends upon the individual anomalies present. Choanal stenosis requires emergency surgical treatment. Short stature may require growth hormone therapy. SMMCI tooth itself is mainly an aesthetic problem, which is ideally managed by combined orthodontic, prosthodontic and oral surgical treatment; alternatively, it can be left untreated.

## Disease name and synonyms

The name originally given to this syndrome by Hall *et al*. [1], "Solitary median maxillary central incisor, short stature, choanal atresia/midnasal stenosis syndrome", is now customarily shortened to the first part of this name: "Solitary median maxillary central incisor syndrome" or SMMCI syndrome, as the other features are not necessarily present in all cases. The use of the full description of the single incisor tooth in the name is important, as it emphasises the unique form and position of this tooth, which is the characteristic and most readily observed feature or trait of the condition.

The early observations of this condition [2-8] merely referred to the congenital and hereditary absence of one central incisor. When the association with short stature was recognised in 1976, the name Monosuperoincisivodontic dwarfism was given by Rappaport *et al*. [9], but it was soon recognised that short stature was not always present in the disorder [10]. The names "single central incisor syndrome" or "single maxillary central incisor" or "single incisor" suggested by other authors [11-15], do not adequately describe the peculiarly formed incisor tooth.

To accurately describe the characteristic tooth present in this syndrome, it is necessary to specify:

• **Solitary**: the tooth present exists as the only central incisor tooth in the maxilla.

• **Median**: this tooth is present precisely in the midline of the maxillary alveolus (a single central incisor tooth present to one or other side of the midline indicates that the contralateral tooth has been lost from trauma or disease, or did not continue to develop beyond the cellular stage, the tooth germ being resorbed).

• **Maxillary**: this characteristic tooth occurs only in the maxilla and not in the mandible.

• **Central Incisor**: the tooth is a central incisor tooth, although of unusual crown form and is not a supernumerary tooth (mesiodens).

Hence, the acronym **SMMCI syndrome**.

## Excluded diseases

The following circumstances where only one central incisor tooth is present are not SMMCI:

• Any condition where two maxillary central incisor teeth commenced development normally, but where one failed to proceed beyond the cellular developmental stage. In this case, the remaining normal tooth develops to one side of the midline, but may erupt in or near the midline.

• Traumatic loss of one central incisor tooth.

• Fusion of a primary and/or permanent central incisor tooth with a supernumerary tooth.

• Mesiodens, which is a supernumerary tooth of conical form, erupting in the midline, but developing to one or other side of the midline in the permanent dentition only.

## Definition

SMMCI syndrome (phenotype) is a unique developmental abnormality, probably a developmental field defect, arising from an unknown event or events occurring between the 35th and 38th days *in utero*, and involving midline structures of the head including the cranial bones, the maxilla and its contained dentition (specifically the central incisor tooth germs), the nasal airways (choanal atresia, midnasal stenosis or congenital pyriform aperture stenosis), and sometimes the brain (holoprosencephaly), together with other midline structures of the body.

## Diagnostic criteria

The frequency figures given below are taken from the published, Royal Children's Hospital (RCH), Melbourne Index Series of cases 1966–1997 [[1], Table 1].

The SMMCI tooth, as with the other features of this syndrome, may possibly occur as an isolated trait [16] (but it is possible that some cases reported as having a solitary incisor as an isolated phenomenon, may not have received a detailed paediatric, ear-nose-throat or genetic examination). It has been found (on review of neonatal paediatric histories in all the RCH series of cases) that one of the three forms of congenital nasal obstruction was present in over 90% of babies. In one other reported case [17], absence of the corpus callosum, congenital pyriform aperture stenosis and the maxillary alveolar and palatal shape in a neonate, lead to the presumptive diagnosis of SMMCI syndrome before the primary SMMCI had erupted.

Diagnosis is possible with ultrasound at 18–22 weeks or, possibly, on genetic testing in familial cases, but is rarely made prenatally. It can certainly be made at birth. In the RCH series, diagnosis varied from 1–9 yrs of age. With the present awareness of the condition, diagnosis should be made no later than 8 months of age on eruption of the primary maxillary incisor tooth.

There is a wide variability in the phenotypic spectrum, even intrafamilial, but the following features are typical of the phenotype:

• Preterm birth and low birth-weight in 37% (diabetic pregnancies in 14% of mothers).

• At birth, a pseudo-notched or arch-shaped appearance of the upper lip with an indistinct philtrum, due to the elevated midline caused by the extremely prominent maxillary alveolus over the developing primary SMMCI tooth and the absence of a labial frenulum together with a narrow nose. The palate is "V"-shaped with an unusual narrow ridge along the midpalatal suture, extending from the incisive papilla to the posterior border of the hard palate (cleft lip and/or cleft palate present in 25% cases) [18,19].

• At birth, potentially life-threatening congenital nasal airway obstruction: either choanal atresia, midnasal stenosis or congenital nasal pyriform aperture stenosis (CNPAS) in over 90% cases. The midnasal stenosis may include septal deviation. The type and degree of severity of the obstruction varies. A recent study of 20 cases of CNPAS found SMMCI in 60% of the patients [20]. CNPAS can be a localised dysostosis without midfacial hypoplasia and with or without SMMCI [21]. Neonates with choanal atresia and CNPAS require surgical intervention. The nose may appear hypoplastic and the nostrils anteverted [see also [22-29]].

• At approximately 8 months age, the eruption of a symmetric solitary maxillary central incisor tooth, situated precisely in the midline of the maxillary alveolus and present in both primary and permanent dentitions (shown on dental radiograph). The symmetry can be confirmed using computer flip imaging. The contour of the two distal surfaces of the SMMCI have the characteristic anatomical shape of the distal surface of a normal central incisor tooth. It can be seen on dental radiographs that the intermaxillary suture is absent. Rarely, asymmetric variations in crown form occur and also rarely, there may be two fused primary central incisors but followed by one typical SMMCI.

• Family history of SMMCI or of holoprosencephaly, microcephaly (or small head circumference), congenital nasal obstruction, very short stature, slow learning or intellectual disability, epilepsy or other midline defects are found in 25% of the cases [30].

• Short stature in 50% cases; potential growth hormone-deficient short stature in only 33% (less than -2 S.D. {standard deviation} from the mean) [see also [30]].

• Small head circumference(in 33%).

• Hypotelorism (in 45%), usually mild, but variable (less than -2 S.D. from the mean interpupillary distance, or less than 3rd centile).

• Holoprosencephaly (HPE) spectrum is the most described association with SMMCI [30]. However, in the RCH series of 25 consecutive cases there have been no cases with HPE. When HPE is present, clinical expression is extremely variable ranging from alobar HPE and cyclopia [31], to microforms of HPE [32-36]. In cyclopia, malformation of the midline structures has been found (suture and cartilage) anterior to the sella turcica [37,38].

• Intellectual disability varying in degree (in 50%): from slow learning (in 25%) to intellectual retardation (in 25%), possibly associated with epilepsy; attention deficit hyperactivity disorder (ADHD) has been found in a high percentage of HPE cases [39].

• Deviant sella turcica and pituitary gland morphology (in 10–50%) [19,37].

• Pituitary gland morphological abnormalities (in 15% of a CNPAS series), found on magnetic resonance imaging (MRI) [20].

In addition, other traits and conditions have been recorded in children with SMMCI. Nanni *et al*. [30] have published a detailed list (with references) of all systemic abnormalities reported in children with SMMCI.

### Association with known syndromes and associations

• CHARGE association (1/21 cases RCH series) [see also [30,40]].

• VACTERL association (2/21 cases RCH series).

• VCF (Velocardiofacial) {del(22)q11.2 syndrome} (1/21 cases RCH series) [see also [30,41]].

• Autosomal dominant HPE.

• Ectodermal Dysplasia [see also [42,43]].

• Duane retraction syndrome [44].

### Association with chromosome abnormalities

• del(18p) [45-47]

• r(18) [48]

• del(7q 36q ter) [49-51]

• 47XXX [52]

• del(22q11.2)

### Association with mutations in the gene SHH

The relevance of the association of SMMCI with these syndromes and chromosome anomalies is still unknown.

### Other abnormalities recorded

• Congenital cardiac abnormalities (in 25%); Tetralogy of Fallot (in 15%).

• Cervical and thoracic spine abnormalities: hemi- and anomalous cervical vertebrae and scoliosis (in 14%); lumbosacral agenesis and anteroposterior split cord malformation [53]; clavicular hypoplasia and peripheral neuropathy [54].

• Other midline abnormalities (in 25%): panhypopituitarism, hypothyroidism, oesophageal and duodenal atresia (in 10%), ambiguous genitalia, micropenis, cervical dermoid and aberrant left subclavian artery [see also [35,46,55-60]].

• Anosmia/hyposmia is rare and difficult to test in young children.

• Various other minor anomalies were recorded in all cases.

General abnormalities (in 50%) included allergies and asthma (20%), multiple haemangiomas, alopecia, absent kidney, alopecia with parchment skin, anal fissures, ptosis, ocular coloboma [61], congenital talipes equinovarus (CTEV), oligodontia, absent thumb and duplication of thumb [44].

Classification of SMMCI is difficult due to the extreme variability in the phenotype and its component features. Kjaer *et al*. [19] suggest classifying SMMCI syndrome cases according to clinical symptoms and craniofacial morphology.

## Differential diagnosis

This is solely one of degree of variability of the different phenotypic features. The SMMCI tooth must be present in all cases and can be detected radiologically prior to its eruption. Eliminating the conditions where only one incisor is present for the reasons given in the exclusion section, there are no other known conditions where this characteristic form of incisor tooth occurs. One of the three forms of nasal obstruction and short stature will be present in most cases. The SMMCI tooth should be considered a predictor of HPE (especially if in addition there is a small head circumference and hypotelorism) when MRI is indicated. In the majority of cases, HPE does occur in the absence of SMMCI.

## Epidemiology

Hall [1] has estimated an incidence of 1:50,000 live births, on the basis of the published Royal Children's Hospital Melbourne Index series, and known cases in other Australian centres (allowing for under-reporting of cases by general dental practitioners and parents). The incidence is higher in stillbirths and aborted foetuses. All cases of cyclopia reported and examined have had a SMMCI tooth [37-39,62]. This incidence for SMMCI is in contrast to one of the three other major traits present in almost all cases of SMMCI syndrome – choanal atresia, which has an incidence of 1:5,000 live births. Holoprosencephaly occurs 1:16,000 live births, but is found in 1:250 spontaneously aborted foetuses [62].

## Clinical description

An infant with SMMCI syndrome will appear normal apart from a small head, hypotelorism and a small narrow nose with an elevated or arched midline of the upper lip giving at times a pseudo-cleft appearance [18]. The infant may have been born preterm with low birth weight and neonatal nasal obstruction, frequently choanal atresia requiring surgical intervention or midnasal stenosis, and less commonly congenital pyriform aperture stenosis. There may be a family history of some or all of the features of SMMCI, especially short stature, neonatal nasal obstruction, breathing difficulty or slow learning.

At 7–8 months age, a solitary median primary central incisor tooth will erupt precisely in the midline of the maxillary alveolus forming a midline prominence. Intraoral examination will reveal absence of the normal midline labial frenulum and a low or normal vaulted palate with a fine bony ridge running the length of the hard palate over the midpalatal suture. Developmentally, the child may be a slow learner or be more severely intellectually delayed. Short stature may be noted early, in some cases requiring growth hormone therapy. Congenital heart disease may be present and usually there will be multiple other minor anomalies, often midline related. Diagnosis may have been made or predicted prenatally or at birth from the characteristic shape of the maxilla, even before the SMMCI tooth erupted.

The simplest case, therefore, will have the facial and oral features described, with perhaps mild midnasal stenosis, slight short stature and slow learning. The average case will have more marked features, with some intellectual disability. The most severely affected cases will have most of the features mentioned above, plus holoprosencephaly or perhaps another syndrome or a chromosome anomaly.

## Management

Management is interdisciplinary. It can be considered in the different specialty areas and chronologically from birth.

Should this anomaly or HPE be suspected from routine prenatal ultrasound, a neonatal paediatrician will normally be present at the birth. In the unsuspected case, at birth, the obstetrician or neonatal paediatrician will diagnose any respiratory difficulty due to congenital nasal obstruction and immediately call a paediatric otolaryngologist to investigate the nasal airways (by nasendoscopy if necessary); if choanal atresia or severe midnasal stenosis is present, a surgical "sound" will be passed through the bony obstruction and a nasopharyngeal tube (or tubes) will be inserted. If CNPAS is present, a plastic surgeon may be required to enlarge the anterior nares and place a "stent".

If (due to the shape of the maxilla combined with narrowing of the nasal airway) the neonatal paediatrician suspects SMMCI syndrome, early referral to a paediatric dentist and to a geneticist will enable the diagnosis to be confirmed and the family pedigree to be researched. No active dental treatment other than preventive care is required for the primary dentition.

The geneticist will assess head circumference and whether hypotelorism is present. If HPE is then considered as a possibility, the geneticist will consult with a paediatric neurologist and an MRI examination of the brain will be undertaken. A developmental paediatrician should monitor the growing child. Should true short stature be suspected, referral to a paediatric endocrinologist is indicated and the need for the administration of growth hormone determined.

Should major or minor anomalies be present in association with SMMCI syndrome, the appropriate paediatric consultants will need to be involved in addition to those mentioned above (a paediatric cardiologist, cardiac surgeon, general surgeon, ophthalmologist, speech pathologist, thoracic physician and allergist, for example).

Not all of the key specialists will need to be involved in every case.

In the simplest case with SMMCI only and mild nasal airway narrowing, all that is required for ideal management (following diagnosis and genetic counselling) is a good paediatric dental care. Facial growth analysis (including transverse facial growth) and photographic series should be included in the regular dental reviews [63]. No treatment is carried out in the primary dentition.

At the appropriate age, in the permanent dentition, the orthodontist will use an appliance to widen the palate, providing sufficient room for the SMMCI tooth to be moved electively to one side of the midline. This provides space for a contralateral artificial central incisor to be placed in the arch by a prosthodontist, either with a single tooth implant (at 17–18 yrs age), or by a bridge (or less desirably a denture). The SMMCI tooth is then recontoured using a labial veneer to create the anatomical form of the appropriate side.

In the more complex case with HPE and/or intellectual disability, or associated with a syndrome such as CHARGE or other anomalies, a large number of specialists will be involved in interdisciplinary care. The aim of dental management will be the same, but difficulties of patient cooperation or compliance may necessitate compromise treatment, since conventional orthodontic management may not be possible and other treatment may have to be carried out under general anaesthesia.

While aesthetically it is considered desirable to restore the dental arch to its normal form with two normally shaped and positioned central incisor teeth, this may not be seen as important or as a priority for some parents, who may elect to leave the SMMCI tooth untreated in the midline.

## Aetiology

The aetiology remains uncertain. The basic structures of the maxilla, including the alveolus with the dental lamina and tooth buds, the labial sulcus and palate have formed normally and appear to have developed normally up to the 35th–38th days *in utero*. The maxillary dental lamina is said to fuse in the midline between days 38–40 *in utero*. For reasons unknown at this time, the normal lateral growth (movement) of the maxillae and orbits, together with the other midline structures in the region (which follows their earlier medial movement) appears to have slowed or ceased, causing the left and right dental laminae to fuse prematurely in the midline, thereby preventing the normal formation of the two tooth germs for the left and right central incisors and their intervening bone (the intermaxillary suture is absent anterior to the incisive fossa – presumably prematurely fused), and soft tissue (including the labial frenulum). A similar process results in fusion of the globes in cyclopia. For a SMMCI tooth to form, composed as it appears to be of the two distal halves of the left and right central incisor teeth, the dental lamina must have fused prematurely in the midline, resulting in apposition and fusion of the forming tooth buds [1,30,64].

HPE is a complex developmental field defect of the forebrain in which the cerebral hemispheres fail to separate into distinct halves [65,68]. The basic defect, the failure of forebrain cleavage, is estimated to occur at or before Streeter Horizon XV (days 35–37 *in utero*) [66], and is postulated to be faulty embryonic interaction between the notochordal plate, the neuroectoderm, the brain-plate and the oral-plate [62]. It is suggested that a short notochordal plate may inhibit lateral movement of the neuroectoderm of the optic anlagen which results in the varying degrees of hypotelorism seen. Hypotelorism occurs when normal lateral growth in the ethmoid region (which is the result of rapid growth of the cerebral hemispheres and occurs from the 35th to the 63rd days *in utero*), fails to follow the earlier medial migration of the eyes. The cause of this defect in embryonic tissue interaction is unknown.

It would seem that this could also provide an adequate explanation for the failure of other midline orofacial and nasal structures to achieve their normal degree of lateral movement at this critical time *in utero*, the timing of the defect resulting in nasal airway stenosis anteriorly CNPAS, centrally (midnasal stenosis including septal pathology) or posteriorly as choanal atresia. Thus, nasal airway stenosis anomalies and SMMCI may represent parts of a developmental field defect in which midface dysostosis is associated with central nervous system (HPE) abnormalities [69].

SMMCI is considered one of the most minimal expressions (microforms) of the HPE spectrum. Deletions on chromosomes 7 and 18 (at 7q36.1 and 18p-) which are in chromosomal regions that harbour HPE genes, have been reported [45-52,67]. At least 12 genetic loci are likely to contain genes implicated in HPE [68]. The relationship of SMMCI to the genes currently implicated in the pathogenesis of HPE (*SHH*; *ZIC2*; *SIX3*; *TGIF *and *DKK1*) is still unclear. Recently, a new *SHH *missense mutation (I111F) was discovered by Nanni *et al*. [30] that may be associated with SMMCI. Marini *et al*. [70] have reported a previously undescribed nonsense mutation in *SHH *at codon 128 (W128X), which caused autosomal dominant HPE (ADHPE). Garavelli *et al*. [71] described a novel *SHH *mutation, Val332Ala, in a child with SMMCI. Hehr *et al*. [72] emphasise the wide phenotypic variability in families with HPE and *SHH *mutation.

Kjellin, in 1999 [73] reported one case with congenital pananterior hypopituitarism, carotid aplasia, CNPAS and SMMCI, postulating that the vascular anomaly may have induced hypopituitarism and the SMMCI anomaly. Hall *et al*. [1] postulated that either an anatomic defect of the adenohypophysis or its vascular supply could have influenced the synthesis and/or release of growth hormone in cases of SMMCI with short stature. Kjaer [19] reported significant deviant morphology in the sella turcica in 50% of 10 children, and demonstrated partial absence of cartilage anterior to the sella turcica in an 18 week foetus with cyclopia and an SMMCI tooth [39].

It is likely that a number of mechanisms can give rise to SMMCI syndrome, some of which may also cause HPE. Cohen [74] states that SMMCI should not be considered a microform of HPE, but as either: (1) an integral component of severe HPE; (2) an anomaly that may occur in conditions unrelated to HPE; (3) a solitary manifestation in some members of a dominantly affected family whose members have variable expressivity to HPE with incomplete penetrance; or (4) (more rarely) as an isolated dominant trait with an *SHH *mutation.

Experimentally, Cole *et al*. [75] report an animal (mouse) model with an absent Ig superfamily member Cdon. Cdon is highly expressed in frontonasal process (FNP), and maxillary processes (MXP) of developing mouse embryos which contain signalling centres for face patterning. Mice homozygous for targeted mutations of *Cdon *show facial defects usually associated with microforms of HPE.

## Diagnostic methods

Recommendations regarding the diagnosis of SMMCI with its associated developmental anomalies have been made by Hall *et al*. [1].

Routine prenatal obstetric sonographic examination (from 16–22 weeks) of the head, face, nose, eyes and anterior palate can predict SMMCI. Should HPE be suspected, prenatal genetic testing from a chorionic villus sample (CVS) for a specific HPE associated genetic mutation may enable a diagnosis to be made.

Neonatal examination will reveal CNPAS or choanal atresia and possibly more severe degrees of midnasal stenosis. Such causes of nasal obstruction are evaluated using nasendoscopy and possibly computed tomography (CT) scan. Examination of the face and oral cavity will reveal the typical maxillary midline alveolar prominence, absent labial frenulum and palatal features. Increasingly, presumptive diagnoses are being made at birth with confidence when the above features are present. Diagnosis can be confirmed by a paediatric dentist clinically and radiologically at 7–8 months of age, once the primary SMMCI tooth has erupted. Early diagnosis, particularly if head circumference is small and hypotelorism present, leads to neurological and MRI examination for possible HPE. The standards for head circumference and interorbital distance are available [76]. Measurements less than 2 or 3 standard deviations from the mean are considered significant for microcephaly and hypotelorism.

Serial measurements of height (and weight), will detect true short stature (< 3 SD from the mean). Endocrinological examination and investigation will then detect whether growth hormone secretion is deficient. Lateral cephalometric orthodontic radiographs often detect an abnormal sella turcica, but the relevance of this finding (or of pituitary gland abnormality found on MRI) to pituitary function is still uncertain.

Finally, referral to, and detailed examination by a geneticist or syndromologist will detect any other minor anomalies present. A pedigree and subsequent examination of available family members will elicit any inherited traits or SMMCI. Chromosome and molecular studies are valuable when appropriate to contribute further to understanding the genesis of this condition.

## Antenatal diagnosis

Routine midtrimester prenatal obstetric ultrasound scan at 18 weeks (with measurements as used for gestational dating) will detect a small head and the position of the orbs and nose. An anterior open-mouth view (when baby swallows or yawns) can show the maxillary alveolar, palatal and nasal anatomy, detecting midline abnormalities especially the prominent alveolus anteriorly and elevated midline of the upper lip. Although examination of the face is not mandatory in most routine sonographic obstetric guidelines, it is increasingly being included in major imaging units [77]. Expert medical and genetic post-ultrasound counselling must be available when an abnormality is discovered or suspected. 3D sonographic scans, when further developed, will greatly simplify the detection of abnormalities of the head, face and mouth.

In one reported case of a mother with a SMMCI tooth and mild hypotelorism, genetic testing with identification of a specific mutation at Codon 128 in *SHH *allowed prenatal diagnosis of HPE in the family [67].

## Genetic counselling

This is important in helping patients and their families to understand what is known about the disorder to date, and to clarify information they may have discovered *via *Internet. The compilation of an extended family pedigree may have brought to light associated previously unrecognised associated anomalies in family members and ancestors. Implications for future pregnancies of parents (and later of the child) can be explained. SMMCI has long been recognised as a risk factor for HPE in the next generation and is considered one of the least severe malformations (or microforms) in the spectrum of malformations seen in ADHPE [25,30]. The actual risk of HPE in the offspring of an individual with SMMCI is unclear at present, but SMMCI must be considered a possible predictor of HPE.

## Unresolved Questions

Further series of cases from other centres are necessary to further define and clarify this syndrome.

The following questions need to be answered:

• What is the genetic basis of the SMMCI phenotype?

• What is the actual defect, which "triggers" the defective process at days 35–38 *in utero*?

• What is the actual mechanism of the formation of the SMMCI tooth?

• What is the aetiology of the short stature and growth-hormone deficient short stature in this condition and the occasional case with hypopituitarism? Could a local intracranial peripituitary vascular anomaly induce the defects in SMMCI phenotype?

• What is the significance, if any, of the anatomical abnormality of the sella turcica and pituitary gland seen on imaging in a small proportion of children with SMMCI with or without HPE? Is this related to the question above?

• What is the relationship with HPE? Why are there not more cases of HPE with SMMCI and more cases of SMMCI with HPE?

• Is there any relationship other than coincidental, of SMMCI with CHARGE association and other syndromes with midline anomalies?

• Why are some cases of SMMCI associated with different levels of congenital nasal obstruction (anteriorly CNPAS, posteriorly choanal atresia or centrally, midnasal stenosis)? Is there a temporal factor here between, say days 35–38 of gestation?
